# Care perspectives: Mothers of children with disabilities in a peri-urban setting in South Africa

**DOI:** 10.4102/ajod.v14i0.1463

**Published:** 2025-03-21

**Authors:** Marcia A. Torres, Chioma O. Ohajunwa

**Affiliations:** 1 Division of Disability and Rehabilitation Studies, Department of Global Health, Faculty of Medicine and Health Sciences, Stellenbosch University, Cape Town, South Africa; 2 Africa Centre for Inclusive Health Management, Faculty of Economic and Management Sciences, Stellenbosch University, Cape Town, South Africa

**Keywords:** children with disabilities, mothers, caregiving, community, families

## Abstract

**Background:**

Within lower- to middle-income countries, mothers of children with disabilities often bear the burden of caregiving for their children, and experience various familial, systemic, structural and sociocultural challenges to the fulfilment of this role.

**Objectives:**

This article discusses the barriers and enablers to caregiving experienced by mothers of children with disabilities living in a peri-urban setting in South Africa.

**Method:**

A qualitative study using in-depth interviews was implemented with six mothers of children with disabilities, recruited through total population sampling. Interviews were conducted in three South African languages – English, isiXhosa and Afrikaans. The interviews were translated, transcribed and analysed thematically.

**Results:**

Key challenges experienced in care giving include poverty, a sense of abandonment and communal stigma. Despite the challenges, the mothers identify spirituality and empathetic healthcare workers as a support for caregiving.

**Conclusion:**

Mothers of children with disabilities experience isolation and stigmatisation, are often alienated from accessing community structures on an equal basis with others, creating a barrier to caregiving for these mothers. An inclusive and targeted approach is needed to raise awareness and create peer support groups for mothers of children with disabilities.

**Contribution:**

A sense of isolation, financial challenges and familial abandonment are significant challenges for mothers of children with disabilities, but they find strength in spirituality. Spiritual belief systems and collaboration with community and spiritual leaders are advocated for ongoing communal support for mothers of children with disabilities. An inclusive, authentic intersectoral collaboration is needed to enhance caregiving capacity for mothers of children with disabilities.

## Introduction

Globally, caregivers of children with disabilities (CWDs) face many challenges. Studies conducted in various parts of the world reflect this narrative (Bahry et al. 2019; Dababnah et al. 2018; Pretorius & Steadman 2018). Lower-to-middle income countries (LMICs) face even more unique, contextually influenced challenges in terms of access to resources and holistic support for caregiving for families of CWDs (Pretorius & Steadman 2018; Zuurmond et al. 2019).

Family caregivers comprise relatives, friends, partners or neighbours who provide assistance, typically unpaid, to someone who has limitations in their physical, mental or cognitive functioning (Schulz et al. 2020:1). Family caregivers provide holistic care — emotional, financial, physical and spiritual care, often forming an entire social system that supports their loved ones with a disability, playing a crucial support role (Schultz et al. 2020; Swartz & Collins 2019).

Studies in various parts of the globe reflect that women and mothers of CWD experience more care challenges (Masefield et al. 2022; Mc Aulliffe et al. 2018; Schultz et al. 2020; Zahaika et al. 2021), also revealing how the female gender is a risk factor within caregiving (Schultz et al. 2020). Therefore, being a female caregiver, can be a challenge, in and of itself, to the care giving experience. Furthermore, certain cultural influences and beliefs perpetuate stigma and blame, on the mothers of CWD (Öztürk & Alemdar 2023; Sevgi & Ayran 2024; Sibel et al. 2012; Tsai et al. 2018).

In many sub-Saharan African contexts, poverty, disability stigma (Adugna et al. 2020; Smythe et al. 2022) the absence of social support (Hussain & Raihan 2022), socioeconomic challenges (Brewer 2018; Kamiya 2021; Njoroge & Murenga 2023), challenges to access to healthcare services (Asa et al. 2021; Adugna et al. 2020; Khan et al. 2020; Kwabena 2021; Schichlindi et al. 2020) are some of the challenges faced by mothers of CWD within the region. Despite these challenges, studies show that some mothers of CWD are developing skills to become activists, support each other and fight for the interests of their children (Ebrahim et al. 2014; Hepperlen et al. 2021).

Alternatively, the capacity to advocate and access resources is often influenced by the context within which the mothers reside.

## Challenges and enablers of caregiving within the South African context

In South Africa, the development of inclusive policies, public disability awareness and sensitivity training has supported (Adugna et al. 2020) and contributed to a positive experience of caregiving generally, but with specific differences based on context.

Eliciting narratives of caregiving experiences of mothers of CWD from all contexts is relevant for targeted interventions and support systems that are contextually relevant for addressing their unique challenges, as well as identifying suitable support. While the experiences of mothers of CWD from many regions of the world have been researched as presented here, this is not the same within the African continent, and even less so within peri-urban South African communities.

This above-stated situation is exacerbated by the residue of South Africa’s apartheid history of racialised healthcare provision; therefore, access to resources that support caregiving has been a challenge within certain contexts, for instance the rural and peri-urban contexts, where poverty is rife (Van der Mark et al. 2019b). Studies on disability experiences have been conducted in other peri-urban areas of Cape Town, but the researcher has not found any study that explored disability and caregiving in Lwandle. Therefore, this study aims to contribute to the knowledge on how mothers of CWD within the peri-urban contexts of Lwandle experience caregiving for their CWD. This is aimed at hopefully supporting targeted support for these mothers. Relatedly, the study will also contribute to the discourse of caregiving within Africa, from the viewpoint of mothers of CWD within a peri-urban setting.

## Methods

The study setting and methodology is presented in this section.

### Setting

Lwandle, a township (peri-urban area) in Strand, is in the Western Cape province of South Africa. This area has different types of mainly low cost housing, including government-funded Reconstruction and Development Programme (RDP) houses, and also shack dwellings. The Ikwezi Clinic is the nearest health facility. There are several churches, crèches, schools and informal businesses in this township, as well as one police station. At the beginning of the 21st century, Lwandle was identified by the well-known Lwandle Migrant Museum — a structure of significance. The museum highlights the experiences of the past migrant labour system and hostel life (Murray et al. 2013), which today still has implications for the people living there. The community health workers (CHWs) of Masincedane Community Service (a non-profit organisation) routinely visit mothers of CWDs in Lwandle to provide support. The idea for this study emanated from conversations held with one of the mother who is a healthcare professional and who managed health programmes in the community. One of the authors is also a mother of a child with a disability, hence the personal interest in this study, beyond the professional interest.

### Research design

A qualitative study was conducted using in-depth interviews, which were analysed thematically. The use of a qualitative methodology assisted the researcher to elicit relevant narratives of the experiences of seeking healthcare access for their children from the participants (Creswell & Poth 2018).

The study population consisted of mothers of CWDs who access healthcare services by Masincedane Community Service in Lwandle. The participants were recruited with the assistance of CHWs. Consent forms were downloaded from the University’s Human Research Ethics website in English, isiXhosa and Afrikaans; thereafter, information specific to the study was included and disseminated accordingly.

### Data gathering

Six mothers of CWDs were interviewed. Four mothers were isiXhosa speaking mothers. All the mothers were living with their children, mostly relying on the grant they received for their children from the government. Only one mother was employed on a part-time basis, and the majority of the other mothers were single mothers, one mother was married and one was a widow. Semi-structured face-to-face interviews were conducted in the homes of the participants. This was the preferred mode of data gathering for most of the participants.

The study population consisted of mothers of CWD who had access to healthcare services provided by Masincedane Community Service in Lwandle, as the place where the research was located. This research was only open to participants who received services from Masincedane Community Service. Other potential participants in the area who did not receive services by this NPO were excluded. The participants were recruited with the assistance of CHWs working for Masincedane Community Service in Lwandle. The CHWs were made aware of the study, after gaining permission from this NPO. With home visits performed routinely in the community, the study population was informed by the CHWs who were known to them about the study and of these six were willing to participate in the study. The initial aim was 10 participants, but consent was received from only 6 mothers; therefore, we went with these 6 mothers who were available and willing to participate. In addition, since the focus of the study was their subjective experience of this phenomena, we were satisfied that saturation was achieved with the 6 participants.

Table 1 presents the demographics of the selected participants.

**TABLE 1:** Participants’ demographics.

Participant names (Pseudonyms)	Participant marital status	Participant employed	Language spoken	Child’s disability	Age of child (years)
Maria	Single	No	isiXhosa	Cerebral Palsy	15
Sophi	Single	No	isiXhosa	Cerebral Palsy	14
Pat	Single	No	isiXhosa	Partial Deafness	12
Ina	Married	No	English	Intellectual Disability	7
Bea	Widowed	Yes	isiXhosa	Blind	6
Hayli	Single	No	English	Hydrocephaly	5

The pilot interview was carried out to further scrutinise the interview tool and ensure that it supports the study intent and focus. After gaining consent, the participants were contacted in order to schedule the interviews in advance at a time and place of their choice with the assistance of the CHW. One participant chose to do the interview at the NPO office, while other participants chose to do the interview in their homes.

Thematic analysis of data was carried out (Braun & Clarke 2006; Creswell & Poth 2018; Maguire & Delahunt 2017) within six, step-by-step procedure for data analysis (Nowell 2017).

Firstly, the researcher cleaned the transcribed data by deleting all irrelevant background sounds during interview as data were transcribed verbatim and read the transcripts while listening to the recording to ensure that nothing was left out. Secondly, the researcher began to identify units of meaning within the transcribed data that respond to the study focus and lifted them out of the transcripts onto an Excel spreadsheet. Thirdly, units of meaning were categorised and given initial codes. Corresponding quotes from the data where the units of meaning were lifted, were colour-coded for reference.

Fourthly, the researcher thereafter analysed all initial recurrent codes from the transcripts and re-grouped them accordingly into sub-themes under appropriate columns. After further assessing the sub-themes, and ongoing immersion with the data, emerging patterns were identified and grouped into themes.

All emerging themes were reviewed again, identifying all outliers in the data, which were re-analysed and either included in an existing theme or new themes were generated as needed. This was done for each transcript first, and then across all transcripts.

Following the aforesaid, all final themes were defined and agreed upon through a debriefing process. To ensure rich data, the researcher included direct quotes from the participants in the findings to provide evidence from the data. The study outcomes were written up in a relevant manner, with the supporting quotes.

### Ethical considerations

Permission for this study was granted by the Health Research Ethics Committee at Stellenbosch University on 28 April 2022 with the ethical clearance number S21/11/247. Participation in the study was voluntary and participants gave informed consent before data collection began. The form was explained to the participants and their questions were addressed by the researcher. Participants were assured of confidentiality of their identity and that pseudonyms would be used when writing the study. The study has been disseminated to the participants, Masincedane Community Service staff and the clinics involved.

## Findings

The study’s findings reveal several challenges and facilitators to caregiving that mothers of CWDs within this study context experience. We begin with the challenges (Table 2).

**TABLE 2:** Challenges and enablers to caregiving for mothers of children with disabilities.

Themes	Sub-themes
**Challenges to caregiving**	
Living in the water	Poor living conditions
Alone and isolated	A sense of abandonment Stigmatisation
I am struggling	Financial challenges
**Enablers of caregiving**	
Spirituality	Finding practical support Spirituality as empowerment and consolation
Supportive attitudes	Helpful healthcare professionals Helpful neighbours and supportive family members

## Challenges to caregiving for mothers of CWDs

### Living in the water

This theme speaks to the unfavourable living conditions of these mothers with their children, with the sub-theme on poor living conditions.

#### Poor living conditions

An example of these harsh conditions is the challenge to gain even the most basic amenities such as adequate housing for them and their children. They are unable to even maintain their shacks, which often leak when it rains. One mother captured this feeling very well by stating that she often feels like she is living in the water. This lack of resources has implications for their capacity to give adequate care to their CWD:

‘And I need, uh, the house. The house, you see, because I’m staying in the water. The water is coming. The water even now it’s wet. The water comes inside.’ (Ina)

‘Like when it rains, I do not feel right because I get rain, I do not have someone that can help me or someone I can go to when in need of something at home. I just stay at home. Those are the things that I deal with.’ (Sophi)

‘Like now that it’s going to rain, I do not have a place inside or covered area like a veranda where I can hang his [*CWD*] clothes and so then he runs out of clothes to wear.’ (Sophi)

The poor living conditions put a lot of stress on the mothers, as it also impedes their capacity to plan and function within their own homes. The lack of resource is a constant challenge to caregiving, and often keeps them inside, in further isolation, which worsens the challenges.

### Alone and isolated

This theme expresses the experience of a lack of support for these mothers nurturing their CWDs and how they feel isolated and lonely in the community.

#### A sense of abandonment

This sub-theme recognises the lonely journey most of the mothers as primary care-givers face with their children. The mothers expressed feeling abandoned by family, with only themselves to rely on:

‘I have not received any help from the family. I do everything myself.’ (Pat)

‘So, there is no one who helps me. No one like, when I do not have money to buy her nappies, that can help me. So, I end up going to loan sharks [*high interest lenders*] so that I can get her nappies because her nappies are expensive, and they are only 40 inside. So those are the challenges I experience.’ (Sophi)

‘One day I will die and I need to know what will happen with my children. Because I do not have family, no mother, aunt, there is no one helping me. My problem is my problem, and it ends there. Only me know what to do with it because now I live with my children. Even the family from my husband’s side do not care for me. Because only my husband loved me in his family.’ (Bea)

#### Stigmatisation

The mothers also experience stigma and negative attitudes from the community members causing further isolation, as highlighted in the following narrative. The attitudes of their neighbours against their CWD increased their burden of care:

‘I am happy, but my husband is not happy.’ (Hayli)

‘There are times where there are challenges when the neighbours get to quarrel then you get people insulting you about your disabled child.’ (Maria)

The next theme that emerged strongly was related to financial struggles that the mothers face.

### Financial challenges

Financial challenges were narrated across all participants. One mother explained how trying to meet the financial demands of raising her child with a disability while battling with the cost of her own health condition is very challenging:

‘I am a mother who is not well, I take high blood pressure, arthritis and diabetes tablets. So I do not work, I am always here at home. So X relies on the R450 child support grant. We also depend on that here at home. From that we have to eat, I buy him clothes, items for school.’ (Pat)

Two of the mothers described how their financial struggles impact healthcare access for their children, because of the cost of transportation. They often rely on the disability grant they receive from the government or save money to be able to access healthcare:

‘When I need to go the clinic, I also struggle there. I need to save money, for instance, I cannot take her to the clinic on the wheelchair. It is far. We use Nomzamo Clinic. So I need to save money for the taxi fare.’ (Maria)

‘My challenge is that, when I take her to the hospital or clinic, I need to drive her. I do not have transport to take her because the wheelchair that she has does not fit in the taxi. I have to uber when I need to give her some rest, I then need to uber for a car that can fit in the wheelchair then going to the clinic I pay. Also when we get there, it [*Uber*] needs to wait for us. I am also struggling because I am not working. I have no source of income except her grant and a grant that I made for having her.’ (Sophi)

The health challenges of these mothers accompanied by financial constraints and transport issues are all highlighted within these findings. These challenges all impact on their capacity to provide the care needed for their CWD.

## Enablers of caregiving

This theme highlights the factors that support caregiving for the mothers of CWDs.

### Spiritual belief systems

The mothers described how their faith made a difference in their lives in these challenging situations.

#### Finding practical support

Spirituality for half of the mothers in this study was a supportive and a positive factor in their lives. It was not only about the internal strength but also about finding practical help within the faith community. One mother stated how someone at church was able to give her a helpful referral:

‘I got in contact with a doctor at Nomzamo through a friend I attended church with who advised that I take him to that doctor. One of the ears could not hear at all, the other could partly hear. Now he was already older about 11–12 years old and was able to tell that this side mama cannot hear and this side I can hear a little bit. So, at Nomzamo they gave me a letter to Tygerberg [*Hospital*] where we got a date booked to take him there. At Tygerberg they then did lots of tests and confirmed that one ear cannot hear and the other partly hears.’ (Pat)

#### Spirituality as empowerment and consolation

Spirituality is seen as an encouragement, so that they do not give up and continue to care for their children. However, even the capacity to always attend church is affected by the need to constantly tend to their child. One of the mother stated the following in this context:

‘Yes, I get to see and feel encouraged by prayer.’ (Maria)

‘I used to go with her when she was still small, put her on my back and just sit with her and just sing at church. So now that she’s grown, I do not afford to go because when I leave her on this wheelchair, I have to stay an hour and keep checking how she’s doing because there is no older person.’ (Sophi)

In addition, the presence of helpful healthcare professionals within the clinic space was a strong facilitator to caregiving.

### Supportive attitudes

This theme speaks of the benefits of having a supportive community that contributes to their capacity to give care to their children.

#### Helpful healthcare professionals

This sub-theme showcases the importance of healthcare professionals in the lives of mothers of CWDs, and how the assistance of these professionals impacts on the lives of the mothers and their capacity to care for their children. The mothers highlight how the positive attitude of the physiotherapist at the clinic they attend meant that they were able to receive help immediately when they arrived at the clinic. This help and kindness are deeply appreciated by the mothers:

‘Yes, I get help immediately as I usually go to the physio. Like when I need to change the chair.’ (Sophi)

‘Recently I like Nomzamo clinic. I never got any problem at Nomzamo clinic. I never had any query. When I get there, I get attended to. I wait like everyone does until I get called and I get satisfied. There is nothing, I can complain about.’ (Bea)

The value of the community health worker in accessing healthcare for their children was highlighted in the following dialogue:

‘She’s a nice lady. Uh, every time if I have a problem, she helped me. She asked me, uh, X, you need something? I said yes. I need something. I have a problem. She said, okay, okay, it’s fine. I’m gonna help you.’ (Ina)

Supportive family and helpful neighbours made a positive difference, as discussed next.

#### Helpful neighbours and supportive family members

Two participants had positive experiences of family members who played an important role in their lives by supporting them to be able to provide adequate care for their CWDs. The following participant has family members who assist and support her in caring for her child with cerebral palsy. This support is an enabling factor influencing her provision of care to her CWD:

‘Ok my family helps at times when I need to go somewhere then someone would come and help with X. Also with feeding.’ (Maria)

Another example is the participant who confirmed financial assistance from her sister:

‘Yes, my sister, is working by, uh, staying there by Free State. She is sending me [*money*].’ (Ina)

The neighbour of a participant assists and enables her with the storage of her food for her family:

‘I do not have a fridge and the microwave since January that they broke. I do not have any other way. When I buy meat, I ask the next-door neighbour to keep for us. It’s not easy. My problem is that, I do not like that people know about what happens in our home. My fridge is a cupboard it’s not a lie.’ (Bea)

This theme highlights the supportive role family and neighbours can play as important enablers of caregiving for mothers of CWDs in the community.

## Discussion

The specific influences of the caregiving experience that emanated from the study for mothers of CWDs are presented and discussed is this section.

### Challenges to caregiving

#### Inherent challenges

There are mental and inherent factors that influence caregiving for mothers of CWDs. These are factors immanent in individuals, their mindsets, and the personal challenges that they have which may act as a challenge to caregiving. These factors form part of the social determinants of health. In 2001, the World Health Organization (WHO) highlighted 10 social co-determinants for health, namely: class; stress; early life; social exclusion; work; unemployment; social support; addiction; food; and transport (Marmot & Wilkonson 2005 in Mc Nair 2017). These overlapping categories determine one’s health and are issues of human rights. Some inherent factors are enablers, while some are challenges to caregiving.

The mothers carry a great deal of anxiety about their home and family, which subsequently impacts their own health and well-being (Gilson et al. 2018). Concurring with the study outcomes, literature highlights the high levels of stress, anxiety and depression caregivers of CWDs experience when compared to other mothers of ordinary developing children (Masefield et al. 2022). Mothers spend a significant amount of time on caregiving and enabling the participation of their children in life on a daily basis (Harris et al. 2022). Caring for a child with special needs is very demanding for the parents (Rani, Gupta & Anand 2022), leaving them constantly exhausted (Ndirangu & Midigo 2019). Caregivers often report that less time is spent on self-care, relaxation, sleep and access to healthcare for themselves (Harris et al. 2022). The mothers in this study often focus on caregiving as their priority, while neglecting themselves. The flip side of ignoring their own need for support is that this will eventually impact on their own capacity to care for their children. The demands placed on these mothers make them quite vulnerable (Gilson et al. 2018). Mothers of CWDs will benefit from mental health literacy training that will facilitate their caregiving process (Gilson et al. 2018) by giving them the required skills to cope with the great demand caregiving puts on them.

#### Financial constraints and unemployment

The mothers of CWDs are financially constrained constantly as it costs more to care for a child with a disability, than a regular child (Jansen-van Vuuren et al. 2022). Social grants they receive are not enough, and they have to go to loan sharks and have to borrow money at exorbitant interest rates, which means that some of them may remain so indebted that they are unable to afford transport to take themselves and their children to the clinics. Research shows that because of the amount of time given to take care of their child with a disability, these mothers struggle to find gainful employment, as they cannot afford daycare. Scott (2018), argues for a reorganisation of the workspace, and current understanding of work, to include and accommodate the specific challenges of caregivers, especially mothers of CWD. In this context, there is a need to rethink of the world of work. To create spaces for informal or negotiated, flexible employment opportunities, and skills development to support the capacity of these mothers to be able to work and earn an income, while taking care of their CWDs.

#### Stigma is still a dominant challenge

The study found that stigma remains one of the biggest challenges for mothers of CWDs in Africa (Jansen-van Vuuren et al. 2022). Stigma comes from family, healthcare professionals, the community among others. Stigma is a complex phenomenon, which coupled with negative attitudes, can lead to social and economic exclusion (Smythe, Adelson & Polack 2020). The authors categorise stigma into ‘anticipated stigma’ (the expectation of encountering stigma), ‘internalised (or self) stigma’ (a sense of shame, guilt and fear) and ‘experienced stigma’ (discrimination). Sources of stigma can include the community, health staff, teachers, laws and policies, and this includes ‘enacted stigma’ (which refers to discrimination) and ‘negative attitudes and prejudice’ perpetuated by others, social processes or structures (Smythe et al. 2020:509). The study outcomes reported on most types of above-stated stigmas identified, aside from the enacted stigma, as there wasn’t a focus on policies. Participants in this study experienced ‘anticipated stigma’ because they often expected to encounter stigma, and one can see how their narratives of ‘experienced stigma’ (discrimination), within their immediate environments could inform these expectations. Literature shows that stigma is still one of the biggest influencers of the disability experience within the continent, and often leading to internalised (or self) stigma’ (a sense of shame, guilt and fear) (Smythe et al. 2020). It is, however, noteworthy that the mothers here, did not display a sense of shame or guilt, but rather fear. Fear of the unknown, fear for the future of their children, after they have passed. Rather than displaying shame or guilt, some of them seemed to have accepted their situation, and rather hoped for some helpful intervention to be able to take care of their children. This acceptance may be informed by their personal spiritual belief systems.

#### A general sense of isolation and the lack of personal and/or familial support systems

Participants refer to staying indoors to avoid dealing with the negative attitudes of neighbours, and the insults and stigmatisation of their CWDs. There is often a sense of abandonment from family as breakdown of the family is very common and isolation from friends and family is experienced (Masefield 2022). Mothers of CWDs experience spousal abuse, with high absenteeism of fathers in their lives, leaving the mothers to become sole carers of their CWDs (Mc Aulliffe et al. 2018). There is a gendered view to the discrimination that these mothers face, that is informed by some pervasive community culture and negative attitudes (Jansen-van Vuuren et al. 2021; Van der Mark et al. 2019b). Mothers are often blamed for the disability their child have (Bani & Lach 2024). The dominant belief system of disability within the African context is still often linked to spiritual and socio-cultural issues, and the mother is often blamed for the situation (Chirwa 2017). This situation informs issues of negative attitudes and subsequent abandonment that follows, facilitating a lack of support and disruption in their lives that often impact on their caregiving capacity or quality of care they give to their children.

## Factors supporting healthcare access

### Spirituality and personal beliefs

A scoping review of 15 African countries, related to the factors that impact the quality of life of families of CWDs within the African context, revealed that there is a strong influence of spirituality as both a positive and negative facilitator of well-being for these families (Jansen-van Vuuren et al. 2022). The study’s outcomes posit spirituality as a positive factor. Mothers who participated in this study say spirituality supports their well-being. Spirituality plays a significant role in the way mothers of CWDs experience the disability of their child. Being aware of, and inculcating the spiritual paradigm, while utilising effective communication methods is vital to the mothers, will help them to support their children more effectively (Smith & Blamires 2022). Mothers of CWDs highlight the role of spirituality in providing them with hope, solace and confidence through the challenges (Yilmaz et al. 2019). In the African context, spirituality is perceived to be crucial to improving the quality of life of CWDs and their families (Jansen Van Vuuren et al. 2021). Spirituality provides purpose (Ohajunwa & Mji 2018) builds internal support and strength for these mothers to continue with their care duties for their children, despite the challenges experienced.

### Inclusive healthcare practices

The inclusive healthcare practices and helpful professionals have great significance for the experience of well-being in the lives CWD and their mothers (Yu et al. 2024). The implementation of inclusive healthcare by health professionals and managers potentially decreases health inequalities.

Inclusive health practices do not only refer to the healthcare professional in the facilities alone but also include the CHWs who often come to the homes to administer healthcare support. Parents of CWDs identify Home and Community Based Services (HCBS) as important to the well-being of their children (Bruton et al. 2024). The HCBS bring healthcare support closer to the mothers, especially as the mothers’ struggle with financial constraints and stigmatisation of their children, rather choosing sometimes, to stay indoors in avoidance. Research from Uganda equally reflects the use of healthcare planners in prioritising at community and health facility levels for the improvement of prevention, management and rehabilitation programmes for CWDs (Katongole 2024). Social workers are also encouraged to integrate their services with health professionals in order to improve care for mothers, and to work closely with policymakers to improve support provided to families with special needs (Jafree & Burhan 2020). Healthcare services as implemented by NPOs such as Masincedane Community Service are also important in the community to support mothers of CWD in their bid to provide the best caregiving to their CWDs.

## Conclusion

Mothers of CWDs are often the primary caregivers or the only caregiver, and certain factors influence their capacity to care for their CWD; the main influencers being poverty and stigma as challenges, and spirituality and inclusive and helpful healthcare professionals as a support system. Although the mothers of CWDs in this study struggled with poverty and financial constraints, the sense of isolation and abandonment caused by stigmatisation is clearly evident. Some mothers stay indoors to avoid dealing with the negative attitudes of neighbours. Hence, disability advocacy is encouraged to continue educating the community about the value of people with disabilities and supporting the rights of CWDs (Hepperlen et al. 2021). One can safely argue that any support given to mothers of CWDs will ultimately enhance their capacity to care for their children.

The mothers carry a lot of anxiety and worry about their children, which creates some inherent challenges that can impact their ability to support their children’s healthcare needs. One recommended way to support mothers is the utilisation of their personal spiritual belief systems to create a community of support. Involving spiritual and religious leaderships in initiatives that focus on non-discrimination and disability-related support will be helpful. A more inclusive community would reduce their sense of isolation and abandonment, enabling them further towards caregiving for their children.

We also advocate for the healthcare system to implement a dual support system that supports mothers of CWDs as well as their children. The focus must be on providing optimal healthcare for mothers together with their CWDs. It should be priority to have a dual care programme that simultaneously assesses the mental health of mothers at community healthcare facilities, whenever they seek medical care for their children. This is because of the heavy burden of stress they carry. The role of CHWs in providing support and education to the mothers of CWDs at their homes on a regular basis is very important to address the isolation and stigma these mothers face in the community.

## Limitations

This research is limited to a small geographical area of Lwandle. Therefore, the findings may not be transferable to other populations from different contexts. Another limitation is that this research was only open to participants who received services from Masincedane Community Service. Other potential participants in the area who did not receive services from this NPO were thus excluded. Because of this being a master’s thesis, the scope of the study and time constraints meant that only a small sample of participants could take part in this study.

## References

[CIT0001] Adugna, M.B., Nabbouh, F., Shehata, S. & Ghahari, S., 2020, ‘Barriers and facilitators to healthcare access for children with disabilities in low and middle income sub-Saharan African countries: A scoping review’, *BMC Health Services Research* 20, 15. 10.1186/s12913-019-4822-6 31906927 PMC6945633

[CIT0002] Asa, G.A., Fauk, N.K., Mwanri, L. & Ward, P.R., 2021, ‘Understanding barriers to the access to healthcare and rehabilitation services: A qualitative study with mothers or female caregivers of children with a disability in Indonesia’, *International Journal of Environmental Research and Public Health* 18(21), 11546. 10.3390/ijerph182111546 34770060 PMC8583444

[CIT0003] Bahry, N.S., Mat, A., Kori, N.L., Ali, A.M., Abdul Munir, Z. & Salleh, M.Z.M., 2019, ‘Challenges faced by Malaysian parents in caregiving of a child with disabilities’, *Global Journal of Business and Social Science Review* 7(2), 118–124. 10.35609/gjbssr.2019.7.2(2)

[CIT0004] Bani Odeh, K. & Lach, L.M., 2024, ‘Barriers to, and facilitators of, education for children with disabilities worldwide: A descriptive review’, *Frontiers in Public Health* 11, 1294849. 10.3389/fpubh.2023.1294849 38292375 PMC10824976

[CIT0005] Brewer, A., 2018, ‘“We were on our own”: Mothers’ experiences navigating the fragmented system of professional care for autism’, *Social Science & Medicine* 215, 61–68. 10.1016/j.socscimed.2018.08.039 30212758

[CIT0006] Bruton, L., Storey, M., Gentile, J., Smith, T.L., Bhatti, P., Davis, M.M. et al., 2024, ‘Access to home-and community-based services for children with disability: Academic institutions’ role and areas for improvement’, *Academic Pediatrics* 24(4), 596–604. 10.1016/j.acap.2023.11.002 37939827 PMC11056305

[CIT0007] Chirwa, M.S., 2017, ‘Experiences of parenting children with disabilities: A qualitative study on the perspectives of mothers of children with disabilities in Zambia’, Doctoral dissertation, University of Warwick.

[CIT0008] Creswell, J.W. & Poth, C.N., 2018, *Qualitative inquiry and research design choosing among five approaches*, 4th edn., Sage, Thousand Oaks, CA.

[CIT0009] Dababnah, S., Shaia, W.E., Campion, K. & Nichols, H.M., 2018, ‘“We had to keep pushing”: Caregivers’ perspectives on autism screening and referral practices of black children in primary care’, *Intellectual and Developmental Disabilities* 56(5), 321–336. 10.1352/1934-9556-56.5.321 30273522

[CIT0010] Ebrahim, H. & Muthukrishna, N., 2014, ‘Motherhood and the disabled child in contexts of early education and care’, *Childhood* 21(3), 369–384. 10.1177/0907568214524233

[CIT0011] Gilson, K.M., Davis, E., Johnson, S., Gains, J., Reddihough, D. & Williams, K., 2018, ‘Mental healthcare needs and preferences for mothers of children with a disability’, *Child: Care, Health and Development* 44(3), 384–391. 10.1111/cch.12556 29430692

[CIT0012] Harris, V., Bourke-Taylor, H.M. & Leo, M., 2022, ‘Healthy mothers healthy families, health promoting activity coaching for mothers of children with a disability: Exploring mothers’ perspectives of programme feasibility’, *Australian Occupational Therapy Journal* 69(6), 662–675. 35633058 10.1111/1440-1630.12814 PMC10083926

[CIT0013] Hepperlen, R.A., Rabaey, P., Ament-Lemke, A. & Manley, H., 2021, ‘Caring for a child with a disability in a Zambian community: A study using photo-elicitation’, *Care, Health and Development* 47(4), 422–434. 10.1111/cch.12851 33470454

[CIT0014] Hussain, M.M. & Raihan, M.M.H., 2022, ‘Disadvantage, discrimination, and despair: Parental experiences of caring for children with disability in Bangladesh’, *Asian Social Work and Policy Review* 16(1), 80–91. 10.1111/aswp.12249

[CIT0015] Jafree, S.R. & Burhan, S.K., 2020, ‘Health challenges of mothers with special needs children in Pakistan and the importance of integrating health social workers’, *Social Work in Health Care* 59(6), 408–429. 32614737 10.1080/00981389.2020.1781738

[CIT0016] Jansen-van Vuuren, J., Nuri, R.P., Nega, A., Batorowicz, B., Lysaght, R. & Aldersey, H.M., 2022, ‘Family quality of life for families of children with disabilities in African contexts: A scoping review’, *Quality of Life Research* 31, 1289–1307. 34537914 10.1007/s11136-021-02994-z

[CIT0017] Kamiya, Y., 2021, ‘Current situation of children with disabilities in low-and middle-income countries’, *Pediatrics International* 63(11), 1277–1281. 10.1111/ped.14904 34197680

[CIT0018] Katongole, S.P., 2024, ‘Coverage of child disability detection, management, and rehabilitation health services in central Uganda’, *EA Health Research Journal* 8(2), 168–179. 10.24248/eahrj.v8i2.778 PMC11407134 39296772

[CIT0019] Khan, G., Isaacs, D., Makoae, M.G., Fluks, L.L., Mokhele, T. & Mokomane, Z., 2020, ‘Service providers’ perceptions of families caring for children with disabilities in resource-poor settings in South Africa’, *Child & Family Social Work* 25(4), 823–831. 10.1111/cfs.12761

[CIT0020] Kwabena, K.J., 2021, ‘Disability and poverty: The plight of children with disabilities in Ghana’, Master’s thesis, NTNU.

[CIT0021] Maguire, M. & Delahunt, B., 2017, ‘Doing a thematic analysis: A practical, step-by-step guide for learning and teaching scholars’, *All Ireland Journal of Teaching and Learning in Higher Education (AISHE-J)* 8(3), 3351–3354.

[CIT0022] Marmot, M. & Wilkinson, R. (eds.), 2005, *Social determinants of health*, Oup Oxford, viewed 03 August 2021, from https://scholar.google.co.za/scholar?.

[CIT0023] Masefield, S.C., Prady, S.L., Sheldon, T.A., Small, N., Jarvis, S. & Pickett, K.E., 2022, ‘The effects of caring for young children with developmental disabilities on mothers’ health and healthcare use: Analysis of primary care data in the born in Bradford Cohort’, *Journal of Developmental and Physical Disabilities* 34(1), 67–87. 10.1007/s10882-021-09789-7

[CIT0024] McAuliffe, T., Thomas, Y., Vaz, S., Falkmer, T. & Cordier, R., 2019, ‘The experiences of mothers of children with autism spectrum disorder: Managing family routines and mothers’ health and wellbeing’, *Australian Occupational Therapy Journal* 66(1), 68–76. 10.1111/1440-1630.12524 30264526

[CIT0025] Murray, N. & Witz, L., 2013, ‘Camp Lwandle: Rehabilitating a migrant labour hostel at the seaside’, *Social Dynamics* 39(1), 51–74. 10.1080/02533952.2013.771491

[CIT0026] Ndirangu, E. & Midigo, R., 2019, ‘Understanding the lived experiences caregiving for children living with disabilities in Mukuru Slums, Kenya; Implications for health and wellness in caregiving’, *International Journal of Health and Biological Sciences* 2(1), 24–31.

[CIT0027] Njoroge, E.N. & Murenga, H.M., 2023, ‘Effect of economic costs of caring for children with intellectual disability on family well-being in Nakuru Municipality, Nakuru County, Kenya’, *Journal of the Kenya National Commission for UNESCO* 3(1), 2958–79991–21. 10.17159/sadj.v79i02.17137

[CIT0028] Nowell, L.S., Norris, J.M., White, D.E. & Moules, N.J., 2017, ‘Thematic analysis: Striving to meet the trustworthiness criteria’, *International Journal of Qualitative Methods* 16(1), n. p. 10.1177/1609406917733847

[CIT0029] Ohajunwa, C. & Mji, G., 2018, ‘The African indigenous lens of understanding spirituality: Reflection on key emerging concepts from a reviewed literature’, *Journal of Religion and Health* 57, 2523–2537. 29909518 10.1007/s10943-018-0652-9

[CIT0030] Öztürk, M. & Alemdar, D.K., 2023, ‘The care burden of mothers of children with disability: Association between family quality of life and fatigue’, *Journal of Pediatric Nursing* 73, e418–e425. 10.1016/j.pedn.2023.10.010 37872058

[CIT0031] Pretorius, C. & Steadman, J., 2018, ‘Barriers and facilitators to caring for a child with cerebral palsy in rural communities of the Western Cape, South Africa’, *Child Care in Practice* 24(4), 413–430. 10.1080/13575279.2017.1347146

[CIT0032] Rani, N., Gupta, M. & Anand, B., 2022, ‘Challenges faced by parents and children with disabilities during COVID-19’, *International Journal of Contemporary Pathology* 8(1), 1–6. 10.37506/ijcpath.v8i1.17833

[CIT0033] Schulz, R., Beach, S.R., Czaja, S.J., Martire, L.M. & Monin, J.K., 2020, ‘Family caregiving for older adults’, *Annual Review of Psychology* 71, 635–659. 10.1146/annurev-psych-010419-050754 PMC7291827 31905111

[CIT0034] Scott, E.K., 2018, ‘Mother-ready jobs: Employment that works for mothers of children with disabilities’, *Journal of Family Issues* 39(9), 2659–2684. 10.1177/0192513X18756927

[CIT0035] Sevgi, G. & Ayran, G., 2024, ‘Investigating the caregiving burden and stress of mothers with children with special needs’, *Journal of Pediatric Nursing* 77, e538–e545. 10.1016/j.pedn.2024.05.020 38834403

[CIT0036] Sibel, E. & Gul, E., 2012, *Difficulties of mothers living with mentally disabled children*, viewed 04 August 2021, from https://pubmed.ncbi.nlm.nih.gov/23862248/. 23862248

[CIT0037] Sichlindi, B.M., Mulunda, H., Chishiba, A. & Simui, F., 2022, *Disablers to access to healthcare services experienced by learners with hearing impairment at Musakanya School in Mpika District*, Research and Scientific Innovation Society (RSIS International), Delhi.

[CIT0038] Smith, M. & Blamires, J., 2022, ‘Mothers’ experience of having a child with cerebral palsy. A systematic review’, *Journal of Pediatric Nursing* 64, 64–73. 10.1016/j.pedn.2022.01.014 35158294

[CIT0039] Smythe, T., Adelson, J. & Polack, S., 2020, ‘Systematic review of interventions for reducing stigma experienced by children with disabilities and their families in low and middle-income countries: State of the evidence’, *Tropical Medicine & International Health* 25(5), 508–524. 10.1111/tmi.13388 32145136

[CIT0040] Smythe, T., Mabhena, T., Murahwi, S., Kujinga, T., Kuper, H. & Rusakaniko, S., 2022, ‘A path toward disability inclusive health in Zimbabwe Part 1: A qualitative study on access to healthcare’, *African Journal of Disability* 11, a990. 10.4102/ajod.v11i0.990 PMC9210151 35747757

[CIT0041] Swartz, K. & Collins, L.G., 2019, ‘Caregiver care’, *American Family Physician* 99(11), 699–706. 31150177

[CIT0042] Tsai, H.W.J., Cebula, K., Liang, S.H. & Fletcher-Watson, S., 2018, ‘Siblings’ experiences of growing up with children with autism in Taiwan and the United Kingdom’, *Research in Developmental Disabilities* 83, 206–216. 10.1016/j.ridd.2018.09.001 30248583

[CIT0043] Van der Mark, E., Conradie, I., Dedding, C. & Broerse, J., 2019, *Exploring adaptation and agency of mothers caring for disabled children in an urban settlement in South Africa: A qualitative study*, viewed 03 August 2021, from https://www.researchgate.net/publication/335444980_Exploring_adaptation_and_agency_of_mothers_caring_for_disabled_children_in_an_urban_settlement_in_South_Africa_A_qualitative_study.

[CIT0044] Van der Mark, E.J., Conradie, I., Dedding, C.W.M. & Broerse, J.E.W., 2019b, ‘Exploring adaptation and agency of mothers caring for disabled children in an urban settlement in South Africa: A qualitative study’, *Women’s Studies International Forum* 76, 102271. 10.1016/j.wsif.2019.102271

[CIT0045] Yilmaz, G., 2019, ‘Spiritual orientation, meaning in life, life satisfaction, and well-being in mothers with disabled children’, *Journal of Religion and Health* 58(6), 2251–2262. 10.1007/s10943-019-00925-4 31595446

[CIT0046] Yu, C., Wong, E., Gignac, J., Walker, M. & Ross, T., 2024, ‘A scoping review of paediatric healthcare built environment experiences and preferences among children with disabilities and their families’, *HERD: Health Environments Research & Design Journal* 17(2), 309–325. 10.1177/19375867231218035 38130020 PMC11080387

[CIT0047] Zahaika, D., Daraweesh, D., Shqerat, S., Arameen, D. & Halaweh, H., 2021, ‘Challenges facing family caregivers of children with disabilities during COVID-19 pandemic in Palestine’, *Journal of Primary Care & Community Health* 12, 21501327211043039. 10.1177/21501327211043039 PMC8404632 34455829

[CIT0048] Zuurmond, M., Nyante, G., Baltussen, M., Seeley, J., Abanga, J., Shakespeare, T. et al., 2019, ‘A support programme for caregivers of children with disabilities in Ghana: Understanding the impact on the wellbeing of caregivers’, *Child: Care, Health and Development* 45(1), 45–53. 10.1111/cch.12618 30259548 PMC7379711

